# Sensory Eye Dominance in Treated Anisometropic Amblyopia

**DOI:** 10.1155/2017/9438072

**Published:** 2017-05-10

**Authors:** Yao Chen, Jiafeng Wang, Hongmei Shi, Xiaoxiao Wang, Lixia Feng

**Affiliations:** ^1^Department of Ophthalmology, The First Affiliated Hospital of Anhui Medical University, Hefei, Anhui, China; ^2^Centers for Biomedical Engineering, University of Science and Technology of China, Hefei, Anhui, China

## Abstract

Amblyopia results from inadequate visual experience during the critical period of visual development. Abnormal binocular interactions are believed to play a critical role in amblyopia. These binocular deficits can often be resolved, owing to the residual visual plasticity in amblyopes. In this study, we quantitatively measured the sensory eye dominance in treated anisometropic amblyopes to determine whether they had fully recovered. Fourteen treated anisometropic amblyopes with normal or corrected to normal visual acuity participated, and their sensory eye dominance was assessed by using a binocular phase combination paradigm. We found that the two eyes were unequal in binocular combination in most (11 out of 14) of our treated anisometropic amblyopes, but none of the controls. We concluded that the treated anisometropic amblyopes, even those with a normal range of visual acuity, exhibited abnormal binocular processing. Our results thus suggest that there is potential for improvement in treated anisometropic amblyopes that may further enhance their binocular visual functioning.

## 1. Introduction

Amblyopia is a common visual disorder that affects 1.6% to 3.5% of the population [1]. Patients with amblyopia normally exhibit abnormal visual processing without any discoverable organic pathological ocular abnormalities, and this abnormality cannot be corrected by glasses [2]. Asymmetric refractive errors between the eyes (i.e., anisometropia) during the critical period of visual maturation (i.e., at ages less than 8 years old) is a widely known cause of anisometropic amblyopia [3]. Patients with anisometropic amblyopia tend to have abnormal monocular visual functions in the amblyopic eye [4], abnormal interocular suppression (i.e., the inhibitory influence of the fixing eye on the amblyopic eye under binocular viewing), as reflected by an abnormal sensory eye dominance, and poor stereopsis [5].

In clinical practice, amblyopia is usually treated with patching therapy, to force the patients' brain to learn to see through the amblyopic eye [6]. This therapy, which is efficient in recovering the monocular visual acuity of the amblyopic eye [7], prevents the two eyes from working together. Because amblyopia is a neurodevelopmental disorder [8] that affects both monocular and binocular visual processing, it is unclear whether the binocular visual deficits recover in clinically treated amblyopes. If not, then neural plasticity targeted at those remaining deficits may be required to recover visual functions.

We set to provide a definitive answer to this question by using a binocular phase combination paradigm [9, 10] to quantitatively assess the sensory eye dominance of treated anisometropic amblyopes, who had normal or corrected-to-normal visual acuity in both eyes, to determine whether their binocular visual systems had fully recovered. Specifically, we ask one question: whether the treated anisometropic amblyopes still have abnormal sensory eye dominance. The binocular phase combination paradigm was developed by Ding and Sperling [9] and has recently been adapted to measure the sensory eye dominance in amblyopia by Huang et al. [10].

To answer this question, we measured the sensory eye dominance of each patient to determine the interocular contrast difference necessary for individuals to achieve balanced binocular viewing in binocular phase combination. Any existing abnormal sensory eye dominance suggests a potential for improvement in treated amblyopes. Most (11 out of 14) of our treated anisometropic amblyopes still exhibited binocular imbalance (our measure of “suppression”), whereas none of the controls were binocularly imbalanced.

## 2. Materials and Methods

### 2.1. Participants

Fourteen treated anisometropic amblyopes, between the ages of 6 and 11 years, (average age: 8.50 ± 1.16 years old), were recruited. The participants had normal or corrected to normal visual acuity in both the previously amblyopic eye and the fellow eye. They were diagnosed with anisometropic amblyopia before treatment, and the detailed clinical information of the treated anisometropia amblyopes, including the refractive errors and visual acuity before and after the treatment, are shown in Table 1. The participants were screened at the ophthalmology practice of the corresponding author LF at the First Affiliated Hospital of Anhui Medical University of China. Another fifteen age-matched (between the ages of 7 and 11 years old) normal subjects were enrolled as the controls. All participants have normal or corrected-to-normal visual acuity, an absence of any ocular or oculomotor abnormalities, and no previous eye surgery. All participants were naïve to the purpose of the experiment.

The study was approved by the Institutional Review Board of Anhui Medical University in China. All observations were performed in accordance with the Declaration of Helsinki before the experiment.

### 2.2. Design and Procedure

#### 2.2.1. Stereopsis Measurement

Stereopsis was tested at a viewing distance of 40 cm in a bright room, using Randot stereotest (Baoshijia, Zhengzhou, China). Red-green glasses were worn over subjects' full refractive correction during the test.

#### 2.2.2. Balance Point Measurement

We used the same set up used by Feng et al. [11] to measure the sensory eye dominance. The experimental procedures were conducted with a PC computer running Matlab (MathWorks Inc., Natick, MA, USA) with Psych Tool Box 3.0.9 extensions. The stimuli were generated by a gamma-corrected LG D2342PY 3D LED screen (LG Life Science, Korea; 1920 × 1080 resolution; refresh rate 60 Hz). The observers were asked to sit at a distance of 1.36 m from the screen and viewed the display dichoptically with their full refractive correction spectacles underneath polarized glasses in a silent and dimly lit space. The luminance of the screen background was 46.2 cd/m^2^ and 18.8 cd/m^2^ through the polar glasses. A chin-forehead rest was provided to minimize head movement.

**Figure 1:**
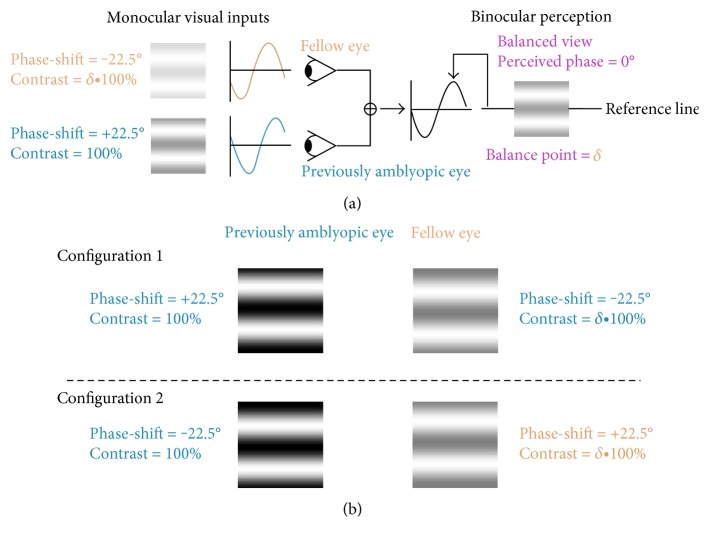
An illustration of the binocular phase combination paradigm for measuring sensory eye dominance. (a) Two horizontal sine-wave gratings with equal and opposite phase-shifts of 22.5° (relative to the center of the screen) were dichoptically presented to observers through polarized glasses; the perceived phase of the cyclopean percept was measured as a function of the interocular contrast ratio; we derived a balance point when the perceive phase was 0°, which represents the interocular contrast ratio at which the contributions of each eye are equal. (b) The phase-shift was +22.5° in the previously amblyopic eye and −22.5° in the fellow eye; similarly, the phase-shift was −22.5° in the previously amblyopic eye and +22.5° in the fellow eye. The perceived phase of the cyclopean grating at each interocular contrast ratio (*δ*) was quantified by half of the difference between the measured perceived phases in these two configurations.

During the test, two horizontal sine-wave gratings (2 degrees × 2 degrees; 1 cycle/deg) with equal and opposite phase-shifts of 22.5° (relative to the center of the screen) were dichoptically presented to observers through the polarized glasses; the perceived phase of the cyclopean percept was measured as a function of the interocular contrast ratio; the contrast of the grating in the nondominant eye was fixed at 100%, and the following interocular contrast ratios were used: 0, 0.1, 0.2, 0.4, 0.8, and 1. We fitted the perceived phases versus interocular contrast ratio (PvR; phase versus ratio curve) curve, and by which, we derived a balance point when the perceived phase was 0°, which represents the interocular contrast ratio when the contributions of each eye are equal (Figure 1(a)). To avoid any potential positional bias, we used two different stimuli compositions in the measurement for each interocular contrast ratio (Figure 1(b)); in one configuration, the phase-shift was +22.5° in the previously amblyopic eye and −22.5° in the fellow eye; similarly, the phase-shift was −22.5° in the previously amblyopic eye and +22.5° in the fellow eye. The perceived phase of the cyclopean grating at each interocular contrast ratio (*δ*) was quantified as half of the difference between the measured perceived phases in these two configurations. Different interocular contrast ratios and configurations were randomized in each trial. We calculated the cyclopean phase and the standard error on the basis of 8 measurement repetitions.

The observers were asked to practice before the experiment to ensure that they understood the task. In each trial, subjects were asked to finish two tasks: eye alignment and phase adjustment. In the line alignment task, they were instructed to move the stimuli (binocular fixation crosses, the high contrast frames, and the monocular fixation dots) in the amblyopic eye to align with the stimuli in the fellow eye. The corresponding coordinate between two eyes was then used in the following phase measurement. The subjects were asked to press the “space” bar on the computer keyboard when they achieved stable vergence. This was followed by a 500 ms presentation of the frames, and then the presentation of two sine-wave gratings in the two eyes, and the observers were asked to finish the phase adjustment task. They were asked to adjust the position of a sided reference line to indicate the perceived phase of the cyclopean grating that they perceived after binocular combination, which was defined as the location of the center of the dark stripe of the grating. The initial position of the reference line was randomly (−9 to 10 pixels) assigned relative to the center of the frame in each trial. The reference line was moved with a fixed step size of 1 pixel, which corresponds to the 4-degree phase angle of the sine-wave grating. The stimuli were presented continually until the subjects finished the phase adjustment task. The observers were asked to press the “space” bar again after they finished the phase adjustment task. The next trial would be started after a 1 sec blank display.

#### 2.2.3. Curve Fits

The perceived phases versus interocular contrast ratio (PvR) curves for different observers were fitted with a modified contrast-gain control model from Huang et al. [10] and Zhou et al. [12]. The fits were conducted in Matlab (MathWorks, Natick, MA) using the nonlinear least squares method.

### 2.3. Statistical Analysis

Two-tailed independent samples *t*-test was used for comparisons between groups. Repeated-measures within-subject ANOVA was used to analyze the relationship between the perceived phase and the interocular contrast ratio. The power and sample size program (version 3.0.43) was used to do the power analysis.

## 3. Results

**Figure 2:**
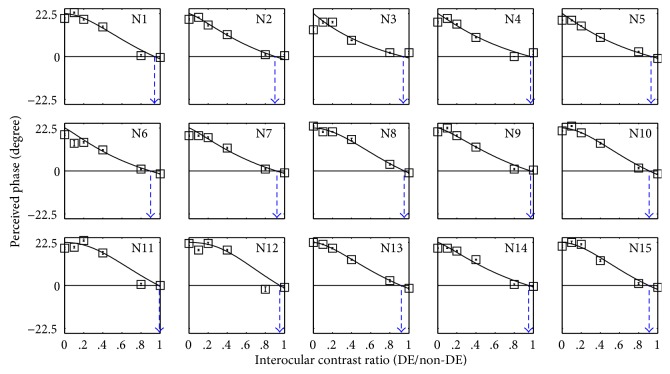
Binocular combination of the normal controls. The relationship between the perceived phase and interocular contrast ratio (dominant eye/nondominant eye) is plotted for 15 normal controls (N1–N15). The crossing of blue dotted line and the horizontal black line represents the balance point at which the two eyes are equally effective. The error bars represent standard errors.

The PvR functions for the normal controls are plotted in Figure 2. The results are consistent with results from previous studies assessing binocular functions in normal controls with the same method [10, 12, 13]. A repeated measures ANOVA indicated that the perceived phase significantly depended on the interocular contrast ratios: *F* (5, 70) = 374.80, *p* < 0.05. The derived balance point from the fitted PvRs (i.e., the interocular contrast ratio where the binocular perceived phase was zero degrees) are marked as triangle symbols in Figure 2, and all the normal observers' balance points were close to 1 (the average balance point of the normal subjects was 0.94 ± 0.03; mean ± SD), thus indicating balanced eyes in the normal controls.

**Figure 3:**
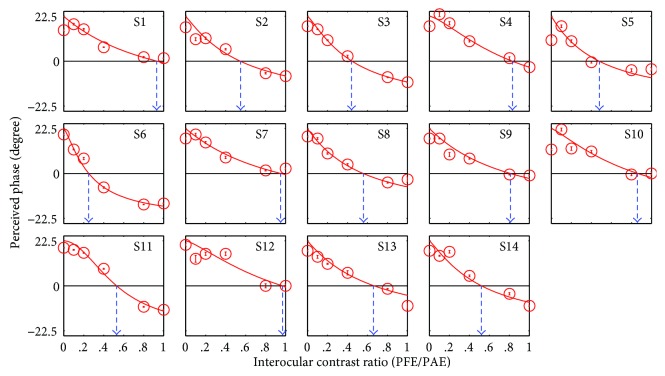
Binocular combination of treated anisometropic amblyopes. The relationship of perceived phase against interocular contrast ratio (previous fellow eye/previously amblyopic eye) is plotted for 14 treated anisometropic amblyopes (S1–S14). The figure is organized in the same manner as Figure 2.

**Figure 4:**
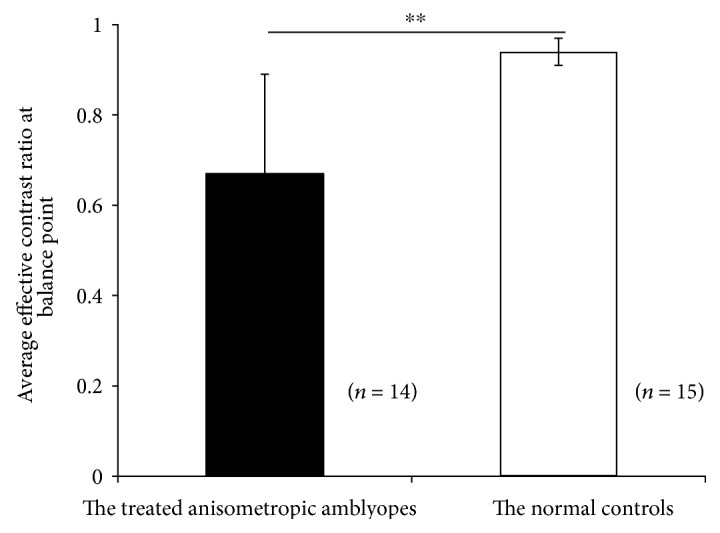
Different sensory eye dominance in treated anisometropia amblyopes and normal controls. The two eyes of the treated anisometropic amblyopes are significantly imbalanced compared with those of normal subjects. “∗∗” represents the result of two-tailed *t*-test for two samples, *p* < 0.05. Error bars represent standard deviation.

**Figure 5:**
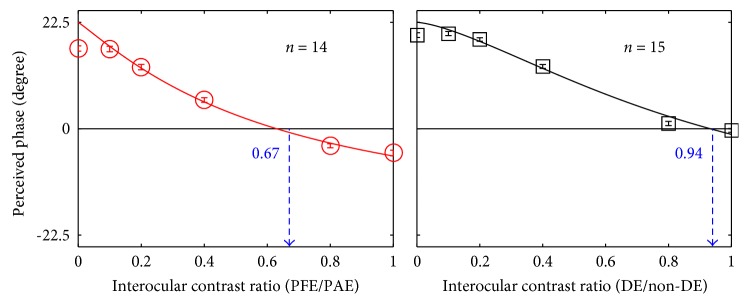
The average PvR curves of treated amblyopia and the controls.

The PvR functions for the treated anisometropic amblyopes are plotted in Figure 3. Similarly, the perceived phase also significantly depended on the interocular contrast ratios: *F* (5, 65) = 112.13, *p* < 0.05. The derived balance points were close to unity in some observers (i.e., S1, S7, and S12). However, most of our treated patients had a relatively small balance point, thus indicating the existence of strong sensory eye imbalance. The average balance point of these treated amblyopes was 0.67 ± 0.22 (mean ± SD), which was significantly different from that of the normal subjects *t* (27) = −4.63, *p* < 0.05, the effect size (using Cohen's *d*) = 1.75, 2-tailed independent samples *t*-test (Figure 4). There was no significant correlation between the degree of anisometropia and the balance point in our treated patients (*p* = 0.12). We also did not find any significant correlation between the balance point and the age at first treatment (*p* = 0.13). In Figure 5, the average PvR curves of the treated amblyopes and the controls are plotted and were found to be consistent with the average balance point shown in Figure 4.

The relationship of average perceived phase against interocular contrast ratio is plotted for the two groups. The figure is organized in the same manner as Figure 2.

## 4. Discussion

In our investigation, we used the binocular phase combination paradigm to quantitatively measure the sensory eye dominance of treated anisometropic amblyopes and found that the contribution of the two eyes were still unequal in most of the treated patients, even though they had normal visual acuity after successful treatments. Only 3 of 14 treated patients showed the balanced pattern seen in normal controls.

Some observers remained different degrees of anisometropia after successful treatment. Recently, Zhou et al. [14] showed that the two eyes are imbalanced in anisometropes without amblyopia, which was not significantly correlated with the degree of anisometropia. In the present study, we also did not find any significant correlation between the degree of anisometropia and the balance point in our treated patients (*p* = 0.12). We suspect that similar mechanisms may account for the abnormal sensory eye dominance in the treated anisometropic amblyopes and the anisometropes without amblyopia.

Using similar methods to those used in this study, we have recently shown that surgically corrected intermittent exotropes also have abnormal sensory eye dominance [11]. Here, we focused on anisometropic amblyopes, whose visual deficits are mechanistically different from amblyopes with strabismus [15, 16]. To the best of our knowledge, this is the first work that shows abnormal sensory eye dominance in treated anisometropic amblyopia. Even abnormal sensory eye dominance has been found in both anisometropic amblyopia and strabismic amblyopia [13], and we do not know whether the abnormal sensory eye dominance we reported here resulted from the same mechanism as that of the surgically corrected intermittent exotropes that we previously reported on [11]. This issue would need to be addressed in future work by using the binocular phase and contrast combination paradigm and the multipathway contrast gain control model [17].

Our study is not the first to show that there is still some degree of visual deficits in treated amblyopes. For example, Huang et al. [18] have also found that treated amblyopes with normal visual acuity remain deficient in contrast sensitivity functions, and the deficits are significant only at high frequencies (i.e., 8 cycle/degree or above). Because our measurements were conducted at a relatively low spatial frequency (i.e., 1 cycle/deg), in which the previously amblyopic eye's contrast sensitivity is normal [18], our results cannot account for the monocular contrast sensitivity deficit of the amblyopic eye. In addition, the contrast of the stimuli in the amblyopic eye was fixed at 100%, which was far above the contrast threshold. Previous reports have found that the amblyopic eye's perception is intact at suprathreshold contrast level [19, 20]. Therefore, the abnormal sensory eye dominance observed herein reflects the learning potential in the binocular processing in treated amblyopes, rather than the monocular contrast sensitivity deficit of the previously amblyopic eye.

Our study provides additional insight into binocular function in treated anisometropic amblyopes. The abnormal sensory eye dominance reported here suggests that the current patching therapy, which can restore the visual acuity of the amblyopic eye, is not sufficient in rebalancing the two eyes in binocular processing. However, our data cannot confirm whether the residual difference in eye dominance has any functional significance, because most of our subjects had normal to near-normal stereopsis. This issue must be addressed in future work. Nevertheless, our results together with those previous reports [18] indicate that the learning (or improving) potential is still present in treated amblyopes, who have normal monocular visual acuity and that additional treatment [21] might be necessary to elicit a “fully” treated status.

## Figures and Tables

**Table 1:** Clinical data of the treated anisometropic amblyopes.

Patient	Age/sex	Best corrected VA (OD/OS)		Stereopsis (arc sec)	Refractive errors (OD/OS)		History
		Before	After		Before	After	
S1	8/F	20/32	20/20	40	+4.75DS/0.75DC^∗^90	+2.25DS/0.50DC^∗^80	Detected at 3 years old, glasses, normal at 6 years old
		20/32	20/20		+2.50DS/1.250DC^∗^105	+1.75DS/1.50DC^∗^100	
S2	8/M	20/80	20/20	60	+5.50DS/1.25DC^∗^100	+3.75DS/1.25DC^∗^100	Detected at 3 years old, glasses, patching, normal at 7 years old
		20/80	20/16		+4.00DS/1.50DC^∗^80	+3.75DS/1.75DC^∗^80	
S3	9/M	20/100	20/20	40	+3.50DS/2.00 DC^∗^90	+1.25DS/2.00 DC^∗^95	Detected at 7 years old, glasses, patching, normal at 8 years old
		20/20	20/16		+1.50DS/1.00DC^∗^90	+1.00DS/1.00DC^∗^85	
S4	8/F	20/32	20/20	100	Plano	Plano	Detected at 3 years old, glasses, patching, normal at 7 years old
		20/40	20/20		+3.00DS	+2.00DS	
S5	8/F	20/80	20/20	100	−4.00DC^∗^180	−4.00DC^∗^180	Detected at 5 years old, glasses, patching, bead-threading, normal at 7 years old
		20/100	20/20		−2.50 DC^∗^180	−3.75 DC^∗^5	
S6	8/M	20/40	20/20	40	+5.25DS	+5.00DS	Detected at 7 years old, glasses, patching, normal at 7 years old
		20/16	20/16		+2.00DS	+1.50DS	
S7	9/F	20/32	20/20	40	+4.50DS/1.00DC^∗^70	+4.25DS/1.00DC^∗^70	Detected at 7 years old, glasses, patching, bead-threading, normal at 8 years old
		20/32	20/20		+7.25DS	+4.50DS	
S8	9/F	20/50	20/20	100	+6.50DS/1.00DC^∗^90	+5.50DS/1.00DC^∗^90	Detected at 5 years old, glasses, bead-threading, normal at 8 years old
		20/46	20/20		+5.25DS/1.25DC^∗^85	+5.00DS/0.75 DC^∗^80	
S9	8/F	20/50	20/25	400	+1.00DS/2.25DC^∗^100	−0.25DS/2.00DC^∗^90	Detected at 6 years old, glasses, patching, bead-threading, normal at 6 years old
		20/32	20/25		1.00DC^∗^100	−0.50DS/0.75DC^∗^90	
S10	9/F	20/40	20/20	100	+4.25DS/0.75 DC^∗^85	+3.00DS/1.00 DC^∗^85	Detected at 5 years old, glasses, patching, normal at 8 years old
		20/32	20/20		+3.00DS/1.00DC^∗^100	+2.75DS/1.00DC^∗^95	
S11	11/M	20/50	20/25	40	+4.25 DC^∗^85	+4.00 DC^∗^95	Detected at 10 years old, glasses, patching, normal at 11 years old
		20/20	20/25		Plano	Plano	
S12	6/M	20/25	20/20	40	+1.00DS/3.50DC^∗^100	+2.50DC^∗^105	Detected at 3 years old, glasses, patching, bead-threading, normal at 5 years old
		20/40	20/20		2.00DC^∗^105	+2.00DC^∗^95	
S13	10/M	20/32	20/16	60	+7.50DS/0.50DC^∗^85	+6.75DS/0.37DC^∗^5	Detected at 6 years old, glasses, patching, normal at 8 years old
		20/40	20/20		+9.00DS/0.50DC^∗^60	+7.00DS/0.37DC^∗^175	
S14	8/M	20/32	20/20	60	+3.75DS/1.00DC^∗^105	+2.50DS/0.75DC^∗^95	Detected at 5 years old, glasses, patching, bead-threading, normal at 6 years old
		20/63	20/20		+2.25DS/1.00DC^∗^90	+2.00DS/1.00DC^∗^85	

VA: visual acuity; OD: right eye; OS: left eye. (i) Visual acuity was measured on the basis of the Snellen visual acuity chart; (ii) all subjects accepted treatment immediately after diagnosis; ∗ represents the axial astigmatism.
